# Exploring Reovirus Plasticity for Improving Its Use as Oncolytic Virus

**DOI:** 10.3390/v8010004

**Published:** 2015-12-24

**Authors:** Vera Kemp, Rob C. Hoeben, Diana J. M. van den Wollenberg

**Affiliations:** Department of Molecular Cell Biology, Leiden University Medical Center, P.O. Box 9600, 2300 RC Leiden, The Netherlands; v.kemp@lumc.nl (V.K.); r.c.hoeben@lumc.nl (R.C.H.)

**Keywords:** virotherapy, oncolytic viruses, oncolytic cell killing mechanisms, combination therapy, mammalian orthoreoviruses

## Abstract

Reoviruses are non-enveloped viruses with a segmented double stranded RNA genome. In humans, they are not associated with serious disease. Human reoviruses exhibit an inherent preference to replicate in tumor cells, which makes them ideally suited for use in oncolytic virotherapies. Their use as anti-cancer agent has been evaluated in several clinical trials, which revealed that intra-tumoral and systemic delivery of reoviruses are well tolerated. Despite evidence of anti-tumor effects, the efficacy of reovirus in anti-cancer monotherapy needs to be further enhanced. The opportunity to treat both the primary tumor as well as metastases makes systemic delivery a preferred administration route. Several pre-clinical studies have been conducted to address the various hurdles connected to systemic delivery of reoviruses. The majority of those studies have been done in tumor-bearing immune-deficient murine models. This thwarts studies on the impact of the contribution of the immune system to the tumor cell eradication. This review focuses on key aspects of the reovirus/host-cell interactions and the methods that are available to modify the virus to alter these interactions. These aspects are discussed with a focus on improving the reovirus’ antitumor efficacy.

## 1. Introduction

**Figure 1:**
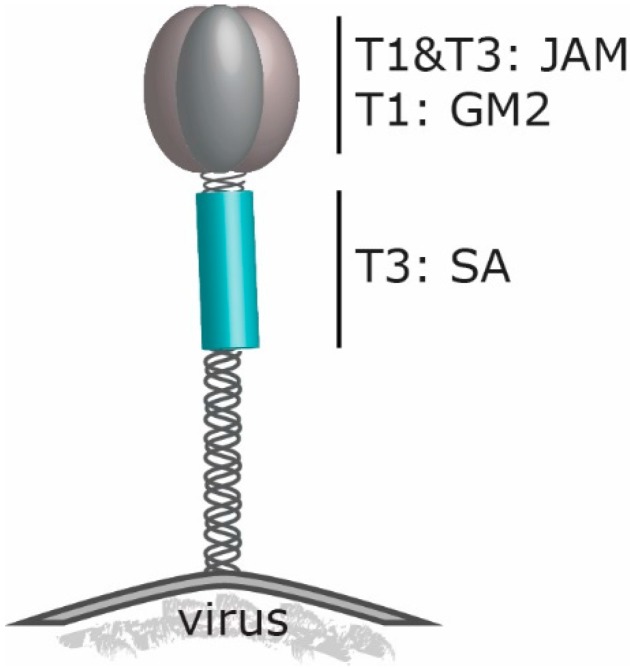
Schematic model of the σ1 trimer at the reovirus capsid. Depicted are the receptor-binding regions of T3D (T3) and T1L (T1). JAM: JAM-A (Junction Adhesion Molecule-A), GM2: ganglioside M2, SA: α2,3; α2,6 and α2,8-linked sialic acid.

The field of oncolytic virus therapy has evolved rapidly since the late 1990s as can be appreciated from the increase in publications on this topic (Figure 1). An overview of viruses currently used in clinical trials for different malignancies is given by Eisenstein *et al.* [1] and Bell *et al.* [2]. The different viruses that are tested can be roughly divided in two groups: (1) wild-type viruses or their attenuated derivatives; and (2) genetically modified viruses containing heterologous transgenes that encode efficacy-enhancing proteins such as cytokines or prodrug-activating enzymes. This review focuses on the use of mammalian orthoreoviruses (reoviruses for short) in oncolytic therapies, and on the various strategies that can be used to enhance their oncolytic potency.

Reoviruses are segmented dsRNA viruses that have not been firmly associated with serious disease in humans. Although reoviruses have been found in children with respiratory and gastrointestinal illnesses, their role remains unclear and there are no convincing data for a causal relation [3] Early on, researchers recognized their capacity to induce cell death in tumor cells, while normal, diploid cells are largely resisting reovirus infection. This observation was first noted in the late 1970s when human cell lines and cell lines from rat, mouse, and monkey origins were exposed to reovirus Type 2 [4]. Most of the more recent clinical studies are carried out with the reovirus Type 3 Dearing (T3D) strain [5,6]. A third reovirus serotype (Type 1 Lang; T1L) is frequently used in comparative studies with reovirus T3D, especially those concerning the mechanisms of infection and replication in cell lines, and the pathogenesis in mouse models [7,8,9]. The classification is based on the difference by the three strains in neutralization and hemagglutinin-inhibition assays [10,11].

Exactly how and why reoviruses prefer to induce cell death in cancer cells has not yet been fully elucidated, despite many studies. A complicating factor here is that many studies reveal only pieces of the puzzle. The variation in responses in different cell lines makes it difficult to combine the results from the various studies. It has been demonstrated that the tumor cell preference of reoviruses can be explained in part by the higher sensitivity of cancer cells with an activated Ras pathway to reovirus-induced apoptosis [12,13,14,15,16]. However, Ras-transformed fibrosarcoma cells (HT1080) can acquire resistance to reovirus-induced cell death. When HT1080 cells are exposed to reovirus T3D, rare cells survive. The reovirus-resistant cells (HTR1) still contain the Ras mutation and are persistently infected by the reovirus. They are resistant to reovirus-induced cell death even after re-infection with a high titer of reoviruses. The parental cells stayed sensitive to reovirus-induced cell death even if they were exposed at a low multiplicity of infection (MOI). In the HTR1 cells, the cathepsin B activity is reduced and this may contribute to the capacity of the reovirus to establish a persistent infection in the cells [17]. For a productive replication cycle leading to lysis of cells, the following aspects are important: (i) attachment and entry into cells; (ii) uncoating by proteases to facilitate escape of the virus from the endosomes; (iii) transcription and replication of viral genomes leading to production of progeny viruses; and (iv) the induction of cell death to release the nascent viruses [18,19,20,21,22]. It has been demonstrated that cathepsin B plays an important role in cell death induction for several viruses [23,24,25]. It remains to be established how the cathepsin B downregulation facilitates the persistent reovirus infection.

One therapeutic approach may not be sufficient to completely eliminate a tumor: this may also be true with oncolytic virotherapies [2]. The heterogeneity of the tumor cells, the presence of therapy-resistant cancer stem cells, and the micro-compartmentalization of the tumor all impede the efficiency of virus entry, replication, and spread, and could lead to the expansion of virus-resistant cell populations. In addition to the heterogeneity within the tumor, there may be heterogeneity between tumors at different locations in the patient, and between tumors in different patients. An example of the latter was provided in cultures of human glioblastoma stem-like cells (GSC). Seven independent serum-free GSC cultures derived from glioblastoma resections were exposed to two different reovirus variants (*i.e.*, wild-type reovirus T3D and the JAM-A-independent *jin-1* mutant, described in [26]). Five parameters were assessed to define the sensitivity of the GSCs to reovirus infection. One of the parameters is the distribution of σ3 protein within spheroids cultures of the GSCs as an indicator of the capacity of the reoviruses to penetrate and spread in the 3D-cell structure. There were large differences in distribution of the reovirus-infected cells between the different GSC spheroids cultures. Also in the monolayer cultures there was a large variation in the infection efficiency, the amount of virus produced per culture and per reovirus-infected cell, as well as in the susceptibility to reovirus induced-cytolysis. These data illustrate the difficulties in establishing proper parameters for predicting the susceptibility of the cells to reovirus-induced oncolysis *in vivo* [27]. These tests only involved *in vitro* cultures and such cultures are obviously not fully representing the clinical tumor *in situ*. In the clinical situation the activity of an active immune system and the tumor microenvironment further add to the heterogeneity of the anti-tumor efficacy of the oncolytic viruses.

## 2. Reovirus’ Engagement to Cell Surface Molecules

Initiation of an infection starts with attachment of the virus to host cells, mostly to cell-surface molecules that are used as receptors. All three reovirus prototype strains can bind with the spike protein σ1 to the canonical reovirus receptor Junction Adhesion Molecule-A (JAM-A). Nevertheless, the three reovirus types differ in their neural tropism in mice [28,29,30]. JAM-A is a cell adhesion molecule that belongs to the tight-junction Ig superfamily. It is involved in cell–cell interactions of epithelial and endothelial cells as well as to leucocytes and platelets. Many of these cell adhesion molecules are exploited by viruses to gain entry into cells. Reovirus strains T1L and T3D are extensively studied with regard to σ1 binding in cell culture systems and the crystal structure of the σ1 complexed to JAM-A has been determined.

Recently, a different protein on cells in the central nervous system (CNS) was identified as a receptor for reovirus, the Nogo receptor NgR1. This is a leucine-rich repeat protein expressed on the cell surface of neurons [31]. Reovirus T3D, but not T1L can infect cultured mouse primary cortical neurons that express NgR1. However, when NgR1 is constitutively expressed in Chinese Hamster Ovary (CHO) cells, not only T3D but also T1L can infect these cells. The precise mechanism for this unexpected observation remains to be established. The difference in glycan binding may route the viruses to different regions in the brain. This may allow T3D to bind to the NgR1 receptor on neurons and T1L to ependymal cells, although more research is required to elucidate the NgR1 pathway in neurons [32].

Most of the receptor studies are done in cells cultured in monolayers [33,34,35]. Monolayer cultures, however, are not representative for tumors when it comes to cell–cell interactions, since the cells are forced to grow on a plastic substrate leaving the apical side exposed to the culture medium and only small areas contact the neighboring cells. In more complex systems, such as in 3D spheroid cell cultures, reoviruses seem to be less dependent on JAM-A for infection. When JAM-A-negative U118-MG cells are grown in spheroids, they become susceptible to wild-type T3D reovirus, whereas the same cells grown in monolayer cultures are fully resistant to reovirus infection. The increased sensitivity to reovirus infection of the cells in spheroid cultures may be related to the high levels of active cathepsin B within the spheroids [36]. The activated cathepsin B promotes the proteolytic uncoating of reovirus particles into intermediate subviral particles (ISVPs). These ISVPs mimic the partially uncoated particles that are formed in endosomes and that penetrate the endosomal membrane to escape into the cell’s cytoplasm. In a similar manner, the ISVPs formed by the action of extracellular cathepsins in spheroids may penetrate the cell membrane independent of a high affinity receptor. This also has implications for the situation *in vivo*, since many cancer types contain increased levels of proteases in their tumor environment (including increased levels of cathepsin B) and this correlates with tumor progression and metastasis [37,38]. Therefore, it remains to be established whether JAM-A expression on cancer cells is an important determinant for reovirus infectivity.

**Figure 2:**
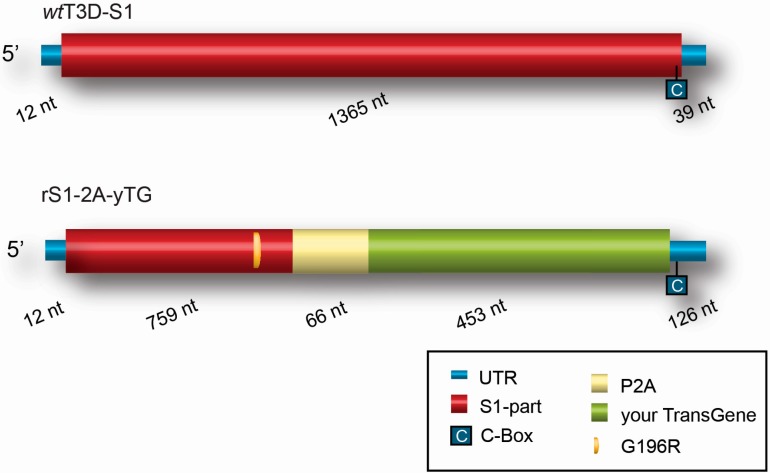
Scheme of recombinant S1 gene segment compared to wild-type T3DS1. rS1-2A-yTG is the recombinant S1 segment containing your transgene (yTG) downstream of a porcine teschovirus-1 element (P2A) to separate the encoded protein from the σ1 part. Entry of the recombinant reovirus by sialic acids is provided by a mutation in the S1-part that results in a G to R amino acid change at position 196 (G196R) in the truncated σ1 protein. In the 3’Untranslated region (UTR) of the recombinant gene the C-box is important for incorporation of the segment in the viral particles [46]. To not exceed the wild-type size of S1, the inserted transgene might be ~453 nt long. The size of each element is depicted in nucleotides (nt) below each segment.

Before reoviruses attach to JAM-A with a high affinity, the viruses engage sialic acids (SA) on the surface of the cells [39]. The sialic-acids binding domain resides in the shaft of the σ1 spike protein. The JAM-A binding domain in the head region of σ1 is more conserved between the different reovirus serotypes [29] than the region binding to sialylated glycans [8,40]. For T3D, the SA binding region is located in the tail part of σ1. In contrast, the domain of T1L binding to ganglioside GM2 has been mapped in the head domain of the spike protein (Figure 2). The difference in carbohydrate binding accounts for the serotype-specific variances in viral spread in murine immune-compromised hosts. In newborn mice, the T1L virus infects ependymal cells and spreads hematogenously causing non-lethal hydrocephalus [40,41]. T3D, however, also infects neurons and uses the neural as well as the hematogenous route for its distribution, leading to lethal encephalitis [42,43,44]. An explanation for the difference in age dependent neural pathogenicity can be explained by the preference for reovirus T3D to infect the unmyelinated CNS of newborn mice in which the NgR1 receptor is not fully associated to myelin and therefore available for reovirus binding, while in adult animals, the NgR1 receptor is myelin-associated, preventing binding to reovirus T3D [32]. This phenomenon had already been reported in 2002 [45]. Taken together, these data demonstrate that the viral spike protein σ1 is a key determinant of viral tropism and spread within the host.

## 3. Reovirus and the Great Escape: What Pathways Are Involved in Reovirus Induced Cell Death

Cell death induction by reovirus is strain-dependent, with T3D strains inducing more efficient cell death than the other strains [7]. T3D reovirus infects neurons and can cause lethal encephalitis, which has been shown to depend on the pro-apoptotic factor Bid [47]. After endosomal escape, but prior to cytoplasmic RNA production, reovirus stimulates essential steps for the induction of apoptosis, which is thought to be the primary mechanism of cell death induced by reovirus infection [48]. The apoptotic pathway is highly complex, and numerous apoptotic factors have been linked to reovirus-induced oncolysis [49,50,51,52,53], making it difficult to identify the trigger that activates the apoptotic cascade induced upon reovirus infection. It has been shown that reovirus-infected cells release soluble TRAIL that induces apoptosis, mainly via death-receptor signaling [54]. In H-RasV12-transformed fibroblasts, reovirus infection inhibits the palmitoylation of Ras, shifting the Ras localization from the plasma membrane to the Golgi system, which eventually stimulates apoptosis. Reovirus replication is favored during Ras localization at the plasma membrane, whereas Ras accumulation in the Golgi enhances apoptosis through increased MEKK1/MKK4/JNK signaling [55]. It seems that the viral σ1 and µL proteins are key determinants in the induction of apoptosis, with σ1 binding to surface receptors and µL facilitating viral disassembly steps [7,56,57,58]. Moreover, the σ1s protein may play a role in cell cycle arrest and the induction of apoptosis [59].

Interestingly, blocking essential components of the apoptotic pathway does not fully diminish reovirus-mediated cell death [60,61,62], prompting the question what other cell-death routes are involved in oncolysis by reovirus. Berger and Danthi described that reovirus T3D, and to a lesser extent T1L, induce RIP1-dependent signaling resulting in necroptosis, a caspase-independent programmed cell-death route resembling necrosis. This route is purportedly activated by either binding to death receptors, pattern-recognition receptors, or via the loss of inhibitors of apoptosis [60]. As for apoptosis, the binding of σ1 to sialic acids affects the induction of necroptosis. However, necroptosis induction relies upon the increased viral gene expression induced by sialic-acid binding rather than the binding itself [63].

Cells undergoing necroptosis often show features of autophagy (*i.e.*, formation of autophagic vesicles) as well, and it remains to be determined whether autophagy is the main mechanism of cell death in necroptotic cells [64]. Autophagy is induced upon infection with various microbial pathogens [65]. It is a highly conserved process involving the degradation of cellular cytoplasmic contents within double-membraned vesicles and recycling of the components in the cytosol, which allows the cell to survive stressful conditions such as a nutrient-poor environment. Many viruses have evolved mechanisms to either suppress or induce autophagy to enhance their replication and/or survival [66]. For avian reovirus, it has been found that the viral p17 protein acts as a nucleoporin Tpr suppressor leading to the activation of p53, p21, and PTEN as well as the repression of the PI3K/Akt/mTOR and ERK pathways stimulating autophagy, and that autophagy induction facilitates virus replication [67,68,69]. Mammalian reovirus induces autophagy in multiple myeloma cells [61,62], but the function of autophagy in these cells remains largely unknown. ER (Endoplasmatic Reticulum) stress can lead to the negative regulation of the mTOR signaling pathway, which in turn initiates autophagy [70]. Interestingly, apoptosis of reovirus-infected multiple myeloma cells has been shown to coincide with ER stress, and ER stress has been implicated to enhance the anti-tumor effects of reovirus, suggesting that autophagy is a potential mechanism of oncolysis by reovirus [71,72,73].

## 4. Reovirus and Its Relation with Neutralizing Antibodies

Many preclinical studies have been done in immune-deficient animals, e.g., to investigate the oncolytic effect in human tumor xenografts in mice [74,75,76]. An attractive route of administration of the oncolytic viruses is intravenous (iv.) infusion since this would allow the infection of tumor (metastases) at multiple sites. In such applications, the presence of preexisting circulating neutralizing antibodies (NABs) directed against the therapeutic virus is considered a major obstacle for an effective delivery [77,78,79]. In the human population most individuals are in their childhood exposed to reoviruses and hence carry NABs [80,81].

However, in a recent study in patients with colorectal cancer metastases in the liver who received one single iv injection with reovirus, replicating reoviruses were detected in certain blood cell compartments (peripheral blood mononuclear cells (PBMCs), granulocytes and platelets) in the circulation, despite the presence of NABs in the blood. Furthermore, after resection of the tumor and surrounding liver tissue, in nine out of the 10 patients, reovirus proteins were detected by immunohistochemistry intra-tumorally and some faint staining in the surrounding tumor stroma or healthy liver tissue. From four patients, freshly resected material was used to make a liver-cell suspension and a tumor-cell suspension to address the question if viable, replicating reoviruses could be detected by a hand-off assay on L929 indicator cells. Plaques were detected in L929 cells exposed to the tumor-cell suspension but not in the L929 cells subjected to the liver-cell suspension. These data suggest that systemically administered reoviruses can reach tumors in patients and preferentially replicate in tumor cells, despite the presence of NABs. The mechanism that is proposed by the investigators is that reoviruses are associated with PMBCs, granulocytes and/or platelets and in that way are protected against the circulating NABs [82].

An earlier study in B16tk melanoma-bearing reovirus-immune mice already showed that *ex vivo* loading of dendritic cells (DCs) or T cells with reovirus could deliver the virus to the melanoma cells *in vivo* after one single iv injection. In this experiment, iv administered reovirus alone, in contrast to the human study, was completely ineffective in killing the B16tk melanoma cells. This corroborates that reovirus hitchhikes on DCs or T cells in a manner that protects them from the preexisting antibodies [83]. In a follow-up experiment, the researchers investigated if cytokine conditioning prior to reovirus injection could enhance the oncolytic effectivity in the mouse model. Their experiments showed that in the presence of preexisting NABs, the addition of granulocyte macrophage colony-stimulating factor (GM-CSF) prior to iv administration of reovirus in B16 melanoma-bearing reovirus-immune mice resulted in significantly reduced tumors and prolonged survival. The proposed mechanism is that reoviruses are transported on GM-CSF-activated monocytes/macrophages into the tumors where the viruses are delivered, start to replicate and destroy the tumor. The associated tumor-cell lysis in turn may activate an anti-tumor immune response [84].

The current dogma dictates that NABs frustrate effective oncolytic virotherapy, but this may not be fully correct. In fact, as the presence of NABs may even enhance the efficacy of virus delivery to the tumor site, the reverse could be true.

## 5. Reovirus and Immune Stimulation

Immunotherapy is an emerging field in the oncology with promising results for cancer patients [85,86,87]. The mission is to achieve lifelong immunity against the cancer in the patient, in order to eradicate the primary tumor as well as distant metastases. Tumors have evolved immune evasion strategies that allow their continued expansion despite active immune surveillance. The strategies include attraction of immune suppressive regulatory T-cells (Treg) and myeloid-derived suppressor cells (MDSCs), the secretion of growth-promoting growth factors and cytokines, as well as secretion of soluble ligands to block tumor-specific effector T-cell recognition and function [88,89]. In recent years, more knowledge has been acquired on the immune cells present in the tumor microenvironment. This results in the development of more therapeutic intervention strategies directed against tumor-associated immunosuppression [90].

The dual action of an oncolytic virus, *i.e.*, preferentially target cancer cells and the strong induction of an anti-viral immune response, may help to inhibit the tumor-associated immune escape. The disruption of immune tolerance may even be more important than the direct oncolytic effect of the virus. In 2009, Prestwich *et al.* [91] showed that reovirus-loaded T-cells did not induce direct oncolysis or virus replication. However, the T-cells could eliminate metastases in lymph nodes and spleen by stimulating anti-tumor immunity. This strategy can be further expanded in combination therapies [92,93]. As mentioned above, the addition of GM-CSF activates and recruits the monocytes/macrophages, which can carry reovirus to the tumor in a mouse model [84]. The addition of GM-CSF in combination with oncolytic viruses is an attractive option. One such approach involves an oncolytic herpes simplex virus that carries a GM-CSF gene as a transgene. This virus is known as Talimogene laherparepvec (T-VEC), previously entitled OncoVex^GM-CSF^ [94]. Promising results of a phase III clinical trial with T-VEC against melanoma have recently been published. In this randomized study, patients with stage IIIB to IV melanoma were treated with subcutaneously administered GM-CSF or intra-lesional injections with T-VEC. In the T-VEC treated group, the durable response rate was improved and the median overall survival was longer compared with the patients receiving GM-CSF alone. The best results were observed in patients with unresected stage IIIB, IIIC or IVM1a melanoma and the treatment was well tolerated with no fatal treatment-related adverse effects [95].

Based on these and other results, T-VEC has been approved by the Food and Drug Administration (FDA) for clinical use in melanoma patients [96] Currently, new melanoma patients are enrolling in a clinical trial combining T-VEC and ipilimumab [97](). Ipilimumab is an antibody directed against T-lymphocyte-associated antigen 4 (CTLA4). The normal biological role of CTLA4, and other molecules involved in immune-checkpoint pathways, is the prevention of autoimmunity by inhibiting T-cell activation directed against “self-ligands” [98,99]. In tumors, this mechanism is hijacked to prevent a tumor-directed T cell response.

Thus far, no data are published on the combination of reovirus and ipilimumab, but preliminary results of studies with another immune-checkpoint receptor, programmed cell death 1 (PD1) seem promising [6]. In a subcutaneous B16 melanoma C57BL/6 mouse model, the combination of anti-PD1 and intra-tumoral administered reovirus enhanced the survival compared to intra-tumoral reovirus alone and the combination achieved a durable anti-tumor response in the surviving animals [100].

Patients with tumors expressing a ligand for PD1 (PDL-1) are the best responders to PD1 and PDL-1 targeted therapies. In some breast, kidney, and lung cancer cell lines, reovirus appears to upregulate PDL-1 expression on the cell surface in the presence of sunitinib. Sunitinib is used as a tumor angiogenesis inhibitor, blocking actions of vascular endothelial growth factor (VEGF). This suggests that a combination of reovirus, anti-PDL-1, and sunitinib would constitute a powerful strategy for some cancers [6,101].

The availability in the clinic of the approved immune-checkpoint inhibitors makes them excellent candidates for the above mentioned combination therapies. However, care must be taken since the underlying mechanisms are not yet fully elucidated. For example, the optimal timing of the regimen still has to be established as suggested by Clements *et al.* [102]. Their study revealed that in the early days after reovirus injection in a murine model for peritoneal carcinomatosis the virus promotes the recruitment of special MDSCs (CD11b^+^, Gr-1^+^, Ly6C^high^) to the tumor microenvironment and thereby transiently (~7 days) induces tumor-associated immunosuppression. In that respect it is interesting to note that in a breast tumor mouse model, colon carcinoma cell and mammary carcinoma bearing mouse models, GM-CSF induces the suppressive MDSCs (CD11b^+^, Gr-1^+^, Ly6C^high^). [103,104]. These data could support the findings by Ilett *et al.* [84] that upon stimulation with GM-CSF in the B16tk melanoma-bearing reovirus-immune mice, CD11b^+^ cells transported the reoviruses to the tumors. The therapy was only effective when GM-CSF preceded the reovirus injections. Their experimental output was obtained after more than seven days, thereby possibly missing the initial induction of tumor-associated immunosuppression.

Another study with vaccinia virus (VV) and CTLA4 inhibition in a syngeneic subcutaneous mouse renal adenocarcinoma model showed that if the combination of virus with anti-CTLA4 was administered on the same day (Day 0) the therapeutic effect of the addition of anti-CTLA4 was diminished. The tumor size decreased when VV was administered at Day 0 and anti-CTLA4 therapy started after four days [105]. In the same report the authors showed that also the used VV strain was of importance. If a VV strain was used in which the gene encoding secreted type I IFN-binding protein (B18R) was deleted, the synergistic effect with anti-CTLA4 was more potent compared to a VV strain that retained the B18R gene. These data suggest that in designing combination therapies the viral strain and timing should be considered especially for viral vectors with immune-stimulating transgenes. This strategy of viro-immunotherapy is an extremely powerful one worth of exploring with oncolytic reoviruses as well.

## 6. Genetic Modification of Reovirus

The RNA genome of the reovirus acquires mutations relatively fast. This provides a plasticity that can be exploited to select reovirus variants that have stronger oncolytic potency. It is also possible to genetically modify the reovirus genome, which offers further options for enhancing the oncolytic activity, for instance by inserting small therapeutic transgenes [46,106]. The recent development of a plasmid-based reverse genetics system [106] for reoviruses provided new options for reovirologists to unveil more aspects of reovirus biology as well as for improving the efficacy in oncolytic reovirus therapy. The powerful reovirus reverse genetics system has primarily been used to resolve issues where reovirus segments are involved in apoptosis, assembly or disassembly, cell tropism and pathogenesis in mice [59,107,108,109,110,111].

Of the studies with the reverse genetics system only a few reports describe its use for generating recombinant reoviruses that harbor heterologous polypeptides in reovirus proteins. The addition of a His(6)-tag to the C-terminus of σ1 to modify the tropism was described in 2008, using a system relying on the expression of one modified segment and infection with a wild-type helper reovirus for providing the other nine gene segments [112]. In 2011 Brochu-Lafontaine *et al.* [113] used the plasmid-based reverse genetics system for adding heterologous polypeptides to σ1. They showed that the addition of a sequence of 750 nucleotides at the C-terminus of σ1 was not tolerated and managed to add a sequence of about 40 nucleotides to σ1. Demidenko *et al.* [114] added longer tandem repeats and inserted a tetra virus 2A element for exogenous polypeptide expression in three other segments (S3, M1 and L1). The total length of heterologous sequences added in the reovirus genome was 1500 nucleotides divided over two segments (L1 and M2). This technique could be useful in the vaccine development and has the possibility to simultaneously express peptides on different segments. The modified reoviruses created were genetically stable for at least three passages in L929 cells. It remains to be seen what will happen if the viruses are propagated for additional rounds on L929 cells.

Another approach to express exogenous proteins in reovirus segments is by replacing an open reading frame (ORF) of a segment for the ORF of the foreign protein. This requires that the viruses are propagated on cell lines that complement for the missing reovirus protein normally expressed from the selected segment [106,115]. Insight was obtained on the various signals in the genomic RNA that are important for replication of the reovirus segments. This allowed for the identification of locations that possibly could harbour transgenes. One such example is the insertion of the small fluorescent protein iLOV (improved Licht, Oxygen or Voltage sensing domain from *Arabidopsis thaliana*) replacing the JAM-A binding region in σ1 of a JAM-A independent *jin* mutant [46]. This confirmed the feasibility of creating autonomously replicating genetically modified reoviruses carrying heterologous transgenes. In Figure 2 the schematic representation visualizes the region in S1 for inserting the transgene and the elements that are provided for entry and separation of the transgene-encoded protein from the σ1 tail in the virus capsid. More research is required to test these recombinant reoviruses in animal models. This platform allows for exploiting the genomic plasticity of reovirus by inserting small genes in the S1 segment to enhance its oncolytic properties.

## 7. Reovirus and Animal Models

The orthoreoviruses are ubiquitous in their geographical distribution and infect many mammalian species including mice, chimpanzees, dogs, cats, cattle, sheep, swine, horses, and monkeys [116]. This indicates that reovirus is able to replicate in different hosts. As in humans, reovirus rarely causes clinical disease in non-human hosts. Upper respiratory or gastrointestinal symptoms are among the possible manifestations of reovirus infection in young and adult animals. It should be noted that in new-born mice, which are immune compromised, reovirus can cause lethal encephalitis, bile-duct atresia, and vasculitis [20]. The pathogenicity of reovirus infection in mice has been reviewed by Montufar-Solis and Klein in 2005 [117].

The incidence of reovirus seropositivity in healthy humans rises from approximately 35% in early childhood, to approximately 60% in teenage years, and more than 85% in late adulthood.

Until now, reovirus has been mainly tested in murine models. Interestingly, the symptoms caused by reovirus infection differ in immune-deficient mice compared to immune-competent mice. Immune-competent mice usually show no severe pathology, whereas in immune-deficient mice the reovirus infection may cause diabetes, or problems with the gastrointestinal, hepatic and central nervous system [118,119,120]. The fact that mice are permissive to reovirus infection allows studying the contribution of the immune system to the eradication of the tumor, as well as the contribution of the immune responses to control the reovirus infections. Kranenburg and collaborators demonstrated that immune suppression promoted antitumor efficacy of reovirus in the murine C26 colorectal cancer model [121].

The wide host range of reoviruses offers attractive options for preclinical research. Various animals such as dogs with spontaneous tumors have been recognized as important models to improve the efficacy of anti-cancer strategies. Since dogs suffer from cancers with a similar disease course as humans, the canine model may mimic the humans more faithfully than xenografted tumors. Several oncolytic viruses are currently being tested for their use in canine patients [111], mostly preclinical-phase research. Canine adenoviruses, for example, have already been used in trials to treat dogs and few side effects were observed. Promising results were reported in a trial for the intra-tumoral treatment of canine melanoma. In this study a replication-deficient adenovirus type 5 (Ad5) vector expressing CD40 ligand was used (AdCD40L) to impair tumor angiogenesis by targeting cells expressing α_v_β_3_ integrin. Nineteen dogs with melanomas (14 oral, four cutaneous, and one conjunctival) were included in the trial and complete disease remission was reported in five of the dogs, eight showed partial remission and four with stable disease. In only two dogs the disease progressed [122]. Taken together, these data demonstrate the feasibility of using spontaneous tumors in companion animals for providing evidence of clinical efficacy before going to clinical trials in humans.

## 8. Future Directions

When the first viruses were discovered, all the attention went to what disease is caused by the virus and what treatment is effective. During the mid-20th century it was discovered that some cancers were caused by viruses [123]. All of these are good reasons to not think of viruses as a solution to cure cancers. With the realization that viruses are very efficient in delivery of genetic material to cells, virologists started exploring viruses as tools.

Reoviruses have the advantage of not being connected to serious human disease and the number of clinical trials that involve the use of reovirotherapy for cancer is still growing. To date, the focus is shifting towards combination strategies, since the efficacy of reovirus as a monotherapy is moderate at best.

In addition to the genetic modification, classical bioselection is another tool that can be used for enhancing the oncolytic properties of reoviruses. It is good to realize that reoviruses have not been evolved as oncolytic agents. Hence the reovirus’ ability to adapt to changing environments may facilitate the selection of more effective variants. One example of such mutants is the *jin* reoviruses. The prolonged propagation of wild-type reovirus on cells that lack JAM-A on the surface forced the virus to develop a strategy to bypass the JAM-A dependency [26]. This principle can be explored for tumor types that resist reovirus infection at other stages of the viral replicative cycle, such as endosomal escape or cell lysis. Some tumors have evolved strategies to evade cell death signaling pathways and resist cancer therapies that rely on triggering apoptosis [124]. For instance, one Ewing sarcoma cell line (STA-ET2.1) that resists most classical anti-cancer therapies, like chemotherapies and radiation treatments [125], also resists death by reovirus [126]. While the j*in-1*, but not wild-type T3D reovirus, can enter the STA-ET2.1 cells and replicate its genome, the replication is not lytic. By continued passaging of the *jin-1* virus on the STA-ET2.1 cells, a mutant was obtained that readily induces cell death in the infected cells [126]). Currently, the mutants are being characterized and evaluated. These mutants may be useful for obtaining more insight into the cell death pathways that are still functional in therapy-resistant cell lines. In this manner the reovirus demonstrates its plasticity and may have a dual use. On the one hand, reoviruses can be used as a potent anti-cancer agent, while, on the other hand, they may help us as probes to study cellular processes.
